# A Chinese patient with the clinical features of Parkinson’s disease contains a single copy of octarepeat deletion in PRNP case report

**DOI:** 10.1080/19336896.2021.1946376

**Published:** 2021-07-05

**Authors:** Qi Shi, Xiao-Jing Shen, Li-Ping Gao, Kang Xiao, Wei Zhou, Yuan Wang, Cao Chen, Xiao-Ping Dong

**Affiliations:** a State Key Laboratory for Infectious Disease Prevention and Control, NHC Key Laboratory of Medical Virology and Viral Diseases, Collaborative Innovation Center for Diagnosis and Treatment of Infectious Diseases (Zhejiang University), National Institute for Viral Disease Control and Prevention, Chinese Center for Disease Control and Prevention, Beijing, China; b Prion Disease department, China Academy of Chinese Medical Sciences, Beijing, China; c Infectious Disease Prevention and control department, Henan Provincial Center for Disease Control and Prevention, Zhengzhou, China; d Center for Global Public Health, Chinese Center for Disease Control and Prevention, Beijing, China; e Chinese Center for Disease Control and Prevention-Wuhan Institute of Virology, Chinese Academy of Sciences Joint Research Center for Emerging Infectious Diseases and Biosafety, Center for Biosafety Mega-Science, Chinese Academy of Sciences, Wuhan, China

**Keywords:** *PRNP*, octapeptide repeat, deletion, Parkinson’s disease, case report

## Abstract

Insertion or deletion of single copy of octapeptide repeat (OR) in human PrP protein are considered as polymorphism, while of insertions of more numbers of OR and deletion of two copies of OR are associated with genetic prion diseases.

Here, we reported a 58-year-old female patient who displayed clinical manifestations of Parkinson’s disease (PD) but contained deletion mutation of single copy of OR in one *PRNP* allele. The patient complained involuntary tremor of left upper limb for 18 months and her symptoms aggravation for 6 months at the time referring to Chinese National CJD surveillance system. The tremor was pronounced at rest, exacerbated by stress and disappear during sleep. Her symptoms were partially relieved after receiving medicament for PD. Neurological examination recorded involuntary movement of left hand and gear-like muscle tension of left upper limb. Coordination movement reported positive of Romberg sign and unstable in heel-keen test. EEG recorded a mild abnormality, but without periodic sharp wave complexes (PSWC). MRI showed a mild write matter demyelination. CSF protein 14-3-3 was negative. *PRNP* sequencing revealed heterozygosity of single copy deletion on ORs (R1-2-3-4/R1-2-2-3-4).

No family history of neurodegenerative disease was recorded. Such case with a single copy of OR deletion in *PRNP* displaying the feature of PD is rarely reported in Chinese mainland.

## Background

Human genetic prion diseases are a group of dominate autosomal hereditary diseases associated with various point mutations or insertion/deletion of octapeptide within PrP protein encoding gene *PRNP* [1–3]. More than 50 different mutations have been described to be disease-associated, displaying clinical phenotypes and neuropathological changes such as Creutzfeldt–Jakob disease (CJD), Gerstmann-Sträussler-Scheinker syndrome (GSS) and Fatal Familial Insomnia (FFI) [1,4,5]. Insertion mutations involve in one to nine (except three) additional octapeptide repeats, while deletion mutations involve one and two octapeptide repeats. Generally, one insertion and one deletion are considered as non-pathogenic genetic polymorphisms, while the rests are pathogenic [3,6,7].

The insertion of additional octarepeats have been reported in Chinese patients of genetic CJD (gCJD) [8], but the deletion of octarepeat(s) is rarely described in Chinese. Here, we reported a woman of 58-year old, who displayed the clinical manifestations of Parkinson disease (PD), contained one octarepeat deletion in her *PRNP* gene.

## Case presentation

A 58-year-old women complaining involuntary tremor of left upper limb for 18 months and the symptoms aggravation for 6 months was hospitalized and referred to China National CJD Surveillance Network. 18 months ago, she started to have involuntary tremor of her left upper limb without definite cause. The tremor was pronounced at rest, exacerbated by stress and disappear during sleep. Occasionally, she had dizziness. She received the therapeutics of improvement for microcirculation and neurotrophic as a suspected Parkinson disease (PD) case when visited to several hospitals, and her symptoms were partially relieved. About 6 months ago, she appeared more symptoms, such as standing unsteadily and moving slowly, and she fell down many times without obvious cause. She complained to have trouble falling asleep and short sleep time. She was hospitalized again with the temporal diagnoses of PD, sleep disorder, hypertension and coronary heart disease.

Her general physical examination did not find significant abnormality. BP: 150/79 mmHg. Neurological examination recorded slow speech and reaction, involuntary movement of left hand, gear-like muscle tension of left upper limb. Coordination movement reported positive of Romberg sign and unstable in heel-keen test, but normal in finger-to-nose and alternating movement test. No notable abnormality was observed in the examinations of cranial nerve and sensory nerve system. Meningeal irritation sign and other pathological reflexes were all negative.

She had hypertension and coronary heart disease for more than 10 years. She denied having diabetes and cerebrovascular disease. Her parents and three other siblings (two brothers and one sister) were healthy. She has a 29-year-old daughter. No family history of neurological and other diseases was noticed.

**Figure 1.**
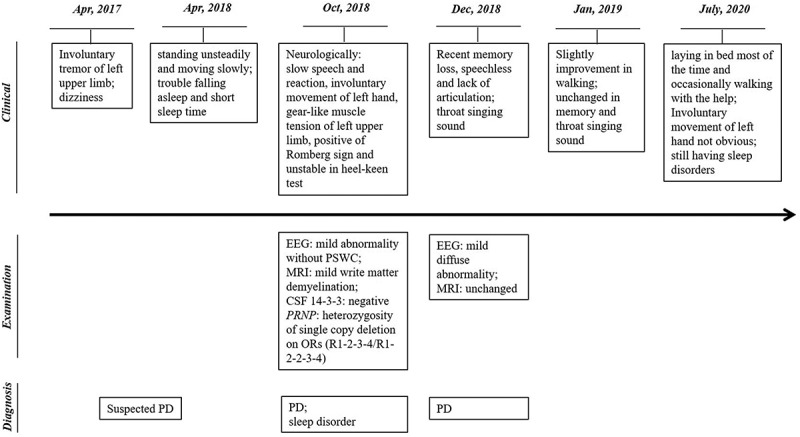
Clinical, laboratory examination and diagnosis information of the patient

Electroencephalogram (EEG) recorded a mild abnormality, but without periodic sharp wave complexes (PSWC). Magnetic Resonance Imaging (MRI) showed a mild write matter demyelination. Lumper puncture was performed and the biochemistry of cerebrospinal fluid (CSF) was in the normal range. CSF protein 14-3-3 was negative by specific Western blot [9](Figure 1).

**Figure 2.**
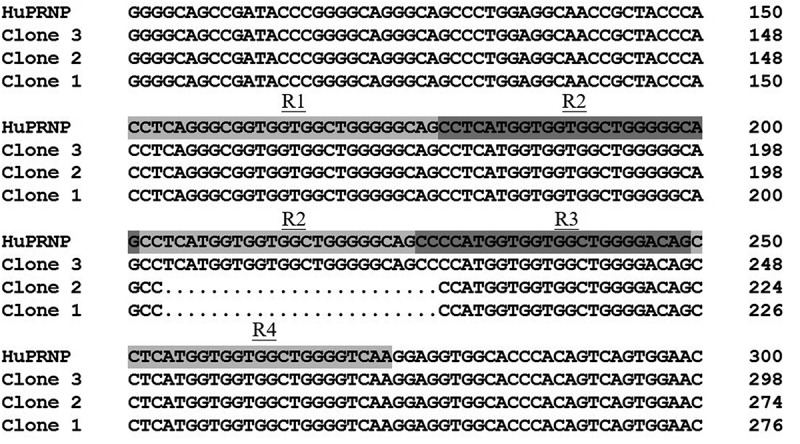
Comparison of the variations of nucleotide of *PRNP* gene of three different clones of the patients with the normal human *PRNP* gene

Her blood was taken and the gene *PRNP* was sequenced after PCR amplification according to the protocol described previously [10]. Surprisingly, scramble signals were observed since nt. 204 of *PRNP*. Further, the PCR product was purified and cloned into a clone vector pT-vector. Sequencing of 10 clones revealed that half of them were wild-type human *PRNP*, the rests were *PRNP* sequence containing a 24-bp deletion from nt. 204 to 227 leading to one octapeptide repeat (OR) deletion. The deletion located in front of the 3^rd^ OR, forming the heterozygosity of single copy deletion on ORs (R1-2-3-4/R1-2-2-3-4) (Figure 2). Additionally, the polymorphism of codon 129 was M/M and that of codon 219 was E/E.

The patient was discharged from the hospital with slight improvement. One week later, she was reported to be rapid recent memory loss, speechless and lack of articulation. Throat singing sound was noticed during sleep at night. She was transferred to another hospital. EEG and MRI were performed again 40 days later, showing mild diffuse abnormality in EEG and almost unchanged in MRI. She discharged from hospital one week later and stayed at home and received the medicament for PD by now. Follow-up one month after discharge recorded slight improvement that she could walk 3 to 4 metres against wall. Her calculating capacity improved somewhat, but her memory, throat singing sound during sleep and other symptoms remained unchanged. Latest follow-up about one and half year later noticed that she was cared at home after last hospitalization. She laid in bed most of the time and occasionally walked and excised with the help of other people. Her memory and calculating capacity did not worse further and she could communicate with others with relatively clear speech. Involuntary movement of left hand was not obvious. There were still sleep disorders but without further aggregation. Her family members refused to take genetic assays.

## Discussion and Conclusions

In this report, we have described a Chinese patient with the clinical manifestations of PD but containing the deletion of one OR in *PRNP*. OR region locates at the N-terminus of PrP and normally consisting of five units is highly conserved among mammalian species [11,12]. ORs possess many biological functions, such as antioxidative activity, binding to divalent ions (copper, manganese, zinc), interacting with proteins, such as tubulin and flotillin-1 [11,13–16]. The insertions of 2, 4 to 9 additional ORs and deletion of 2 units are proved to be closely associated with genetic CJD, while insertion and deletion of one OR are considered as polymorphism [2,6]. Early studies had showed that the deletion of one repeat was common, ranging from 1 to 2.5% in the general population [7,17]. However, such mutation seems to be not very commonly observed in Chinese patients with the suspected diagnosis of prion disease or CJD. Since 2006 we conducted national CJD surveillance network, more than 5,000 cases were referred to the network and more 2,000 various subtypes of prion diseases were diagnosed. Only two cases are confirmed by sequencing to have one OR deletion in their *PRNP*, regardless of prion disease or other neurological diseases. One is the case in this study, the other is a patient displaying typical sCJD manifestations with one OR deletion and a G114V mutation (Guo et al, in preparation). In addition, only two Chinese cases having insertion mutation of additional ORs (one with 1 and the other with 7 units) were identified [10]. Compared with the frequencies of other *PRNP* mutations in Chinese, the mutations within OR region among the cases with prion diseases are less common.

We consider this case as PD rather than a genetic CJD, as she appears slow movement, involuntary movement of left hand, gear-like muscle tension of left upper limb, but without the sign of cerebellar ataxia, progressive supranuclear palsy or cortical synaesthesia loss. Her situation shows different degree of improvement after receiving medicaments for PD. Generally, the patients with small numbers of OR insertion (four or fewer) tends to present as gCJD while those with more numbers of OR insertion usually appear as GSS [2–4,18]. In the period of more than 4-year long after onset of symptoms of this patient, EEG and MRI examinations have repeated. No CJD-associated abnormality has been recorded. It indicates a very low probability of CJD. From the other point, it reflects that the deletion of one OR in this Chinese patient is non-pathogenic, or at least very low penetration.

Variation and polymorphism of *PRNP* have been analysed in the patients with PD. No association of genotypes in codon 129 with the susceptibility to idiopathic PD (IPD) has been figured out in a study covering 400 patients of IPD and roughly same amount of control in the United states [19] and similar study in Germany [20]. In the other study with 528 subjects in Chinese Taiwan [21], no difference in the distribution of codon 129 M/M is found between the groups of PD and control. However, they had found three PD patients with single copy deletion of OR. Interestingly, the location of the deleted OR of the patients in this study is exactly the same as the three Chinese Taiwan PD patients. Two different types of single copy OR deletion have been also observed in a family with familial AD, in which some of the affected family members showed the features of PD, even one was autopsy‐verified AD and PD [22]. Although one OR deletion in *PRNP* is non-pathogenic, further exploration of this polymorphism in the distribution and possible role in neurodegeneration diseases, such as PD, is deserved.

## Data Availability

All data generated or analysed during this study are included in this published article.

## Abbreviations

PD: Parkinson’s disease
CJD: Creutzfeldt–Jakob disease
OR: octapeptide repeat
PSWC: periodic sharp wave complexes
EEG: Electroencephalogram
MRI: Magnetic Resonance Imaging
GSS: Gerstmann-Sträussler-Scheinker syndrome
FFI: Fatal Familial Insomnia

## References

[1] Chen C, Dong XP. Epidemiological characteristics of human prion diseases. Infect Dis Poverty. 2016;5(1):47. 27251305 10.1186/s40249-016-0143-8 PMC4890484

[2] Baldwin KJ, Correll CM. Prion disease. Semin Neurol. 2019;39(4):428–439. 31533183 10.1055/s-0039-1687841

[3] Kovacs GG, Puopolo M, Ladogana A, et al. Genetic prion disease: the EUROCJD experience. Hum Genet. 2005;118(2):166–174. . 16187142 10.1007/s00439-005-0020-1

[4] Jeong BH, Kim YS. Genetic studies in human prion diseases. J Korean Med Sci. 2014;29(5):623–632. 24851016 10.3346/jkms.2014.29.5.623 PMC4024956

[5] Schmitz M, Dittmar K, Llorens F, et al. Hereditary human prion diseases: an update. Mol Neurobiol. 2017;54(6):4138–4149. . 27324792 10.1007/s12035-016-9918-y

[6] Beck JA, Mead S, Campbell TA, et al. Two-octapeptide repeat deletion of prion protein associated with rapidly progressive dementia. Neurology. 2001;57(2):354–356. . 11468331 10.1212/wnl.57.2.354

[7] Windl O, Giese A, Schulz-Schaeffer W, et al. Molecular genetics of human prion diseases in Germany. Hum Genet. 1999;105(3):244–252. . 10987652 10.1007/s004399900124

[8] Wang XF, Guo YJ, Zhang BY, et al. Creutzfeldt-Jakob disease in a Chinese patient with a novel seven extra-repeat insertion in PRNP. J Neurol Neurosurg Psychiatry. 2007;78(2):201–203. . 17229753 10.1136/jnnp.2006.09433 PMC2077666

[9] Gao C, Shi Q, Tian C, et al. The epidemiological, clinical, and laboratory features of sporadic Creutzfeldt-Jakob disease patients in China: surveillance data from 2006 to 2010. PLoS One. 2011;6(8):e24231. . 21904617 10.1371/journal.pone.0024231 PMC3164193

[10] Shi Q, Zhou W, Chen C, et al. The features of genetic prion diseases based on Chinese surveillance program. PLoS One. 2015;10(10):e0139552. . 26488179 10.1371/journal.pone.0139552 PMC4619501

[11] Salzano G, Giachin G, Legname G. Structural consequences of copper binding to the prion protein. Cells. 2019;8(8). DOI:10.3390/cells8080770 PMC6721516 31349611

[12] Gill AC, Castle AR. The cellular and pathologic prion protein. Handb Clin Neurol. 2018;153:21–44. 29887138 10.1016/B978-0-444-63945-5.00002-7

[13] Sanchez-Lopez C, Rossetti G, Quintanar L, et al. Structural determinants of the prion protein N-terminus and its adducts with copper ions. Int J Mol Sci. 2018;20(1):18. . 10.3390/ijms20010018 PMC6337743 30577569

[14] Dong C-F, Shi S, Wang X-F, et al. The N-terminus of PrP is responsible for interacting with tubulin and fCJD related PrP mutants possess stronger inhibitive effect on microtubule assembly in vitro. Arch Biochem Biophys. 2008;470(1):83–92. . 18037369 10.1016/j.abb.2007.11.007

[15] Ren K, Gao C, Zhang J, et al. Flotillin-1 mediates PrPc endocytosis in the cultured cells during Cu(2)(+) stimulation through molecular interaction. Mol Neurobiol. 2013;48(3):631–646. . 23625312 10.1007/s12035-013-8452-4

[16] Li X-L, Dong C-F, Wang G-R, et al. Manganese-induced changes of the biochemical characteristics of the recombinant wild-type and mutant PrPs. Med Microbiol Immunol. 2009;198(4):239–245. . 19633867 10.1007/s00430-009-0120-y

[17] Palmer MS, Mahal SP, Campbell TA, et al. Deletions in the prion protein gene are not associated with CJD. Hum Mol Genet. 1993;2(5):541–544. . 8100163 10.1093/hmg/2.5.541

[18] Bernardi L, Bruni AC. Mutations in prion protein gene: pathogenic mechanisms in C-terminal vs. N-terminal domain, a review. Int J Mol Sci. 2019;20(14). DOI:10.3390/ijms20143606 PMC6678283 31340582

[19] Scholz SW, Xiromerisiou G, Fung HC, et al. The human prion gene M129V polymorphism is not associated with idiopathic Parkinson’s disease in three distinct populations. Neurosci Lett. 2006;395(3):227–229. . 16298483 10.1016/j.neulet.2005.10.081

[20] Gossrau G, Herting B, Möckel S, et al. Analysis of the polymorphic prion protein gene codon 129 in idiopathic Parkinson’s disease. J Neural Transm (Vienna). 2006;113(3):331–337. . 15997418 10.1007/s00702-005-0329-x

[21] Wang V, Chuang TC, Soong BW, et al. Octarepeat changes of prion protein in Parkinson’s disease. Parkinsonism Relat Disord. 2009;15(1):53–58. . 18455951 10.1016/j.parkreldis.2008.03.003

[22] Perry RT, Go RCP, Harrell LE, et al. SSCP analysis and sequencing of the human prion protein gene (PRNP) detects two different 24 bp deletions in an atypical Alzheimer’s disease family. Am J Med Genet. 1995;60(1):12–18. . 7485229 10.1002/ajmg.1320600104

